# Altered white matter network topology is associated with more profound language impairment in children with autism spectrum disorder

**DOI:** 10.1016/j.nicl.2026.104042

**Published:** 2026-07-27

**Authors:** Conghui Su, Zhaoran Liu, Shuiqun Zhang, Aiwen Yi, Yaqiong Xiao

**Affiliations:** a Faculty of Life and Health Sciences, Shenzhen University of Advanced Technology, Shenzhen 518000, China; b Radiology Department, Third Affiliated Hospital of Guangzhou Medical University, Guangzhou 510530, China; c Department of Pediatrics, The Third Affiliated Hospital of Guangzhou Medical University, Guangzhou 510530, China; d Guangdong Provincial Key Laboratory of Major Obstetric Diseases, Guangzhou 510530, China; e Guangdong Provincial Clinical Research Center for Obstetrics and Gynecology, Guangzhou 510530, China; f Guangdong-Hong Kong-Macao Greater Bay Area Higher Education Laboratory of Maternal-Fetal Joint Medicine, Guangzhou 510530, China; g Guangdong Women and Children Hospital, Southern University of Science and Technology, Guangzhou 511400, China

**Keywords:** Autism spectrum disorder, Young children, Language abilities, Graph theory, Brain topological properties, Moderation analysis

## Abstract

Children with autism spectrum disorder (ASD) often exhibit language deficits, yet the influence of varying language deficits on global white matter networks remains underexplored.

In this study, diffusion-weighted imaging data were collected from a cohort of Chinese children with ASD (*n* = 67, 54 boys) and typically developing (TD) children (*n* = 36, 23 boys) aged 20 to 93 months. K-means clustering divided the ASD sample into higher language (ASD-HL, *n* = 29) and lower language (ASD-LL, *n* = 38) subgroups. We examined topological properties of brain networks and compared global and nodal characteristics across ASD subgroups and TD children. Relationships between autism symptom severity, language abilities, and brain network characteristics were also assessed.

The ASD-LL subgroup showed reduced global efficiency (Eg), fewer hubs, decreased fiber connectivity, and fewer inter-hemispheric connections, compared to both ASD-HL and TD groups. Decreased Eg was associated with more severe autism symptoms in the ASD-LL subgroup, but not in the ASD-HL subgroup. Moderation analysis revealed that language ability significantly moderated the link between symptom severity and Eg: higher symptom severity was significantly associated with lower Eg in children with lower language ability, but not in those with higher language ability.

These findings suggest that language deficits contribute to alterations in white matter networks and may modulate the impact of autism symptoms on brain structural inefficiency in children with ASD. This study underscores the importance of targeting language skills in interventions and using language ability as a key stratification factor in ASD research.

## Highlights

• Language stratifies distinct white matter network phenotypes in children with ASD.
• Children with ASD and lower language show disrupted white matter network topology.
• Language moderates the link between autism severity and global network efficiency.
• White matter network alterations in ASD are specifically related to language.
• Language may serve as a meaningful stratification factor for brain studies in ASD.

## 1 Introduction

Autism spectrum disorder (ASD) is a neurodevelopmental disorder characterized by deficits in social skills and the presence of restricted and repetitive behaviors or interests (Lord et al., 2018). The great heterogeneity in its clinical manifestations and behavioral profiles poses major challenges for early diagnosis and effective intervention. Unveiling the neurobiological basis underlying this heterogeneity and identifying the potential brain markers have therefore been a key focus of contemporary neuroscience (Ecker et al., 2015). As ASD follows a developmental trajectory (Fountain et al., 2023; Szatmari et al., 2015), childhood represents a crucial window for characterizing atypical brain development and identifying early biological signatures of the disorder.

The investigation of alterations in brain structure has been paramount, as structural connections reveal the physical architecture that supports and constrains brain functions (Mišić et al., 2016; Sporns, 2013). White matter, in particular, plays a crucial role in shaping functional synchronization and supporting early cognitive development (Feng et al., 2023). For example, greater microstructural integrity of white matter tracts during the first year of life was associated with better cognitive and language outcomes at ages 1 and 2 (Girault et al., 2019). Previous studies have consistently found that individuals with ASD exhibit altered brain structure compared to healthy controls (Cheng et al., 2010; Guo et al., 2024; Postema et al., 2019). However, these studies reported primarily on localized changes, such as the integrity of specific tracts or abnormalities in regional gray matter volumes, rather than examining the system-level organization. The brain, however, functions as an integrated network rather than a collection of isolated regions. Emerging evidence suggests that atypical structural patterns in ASD span multiple brain systems (M. Li et al., 2022; Sha et al., 2022), underscoring the importance of using a network-based approach to explore the neurobiological basis of ASD. Modelling the brain as a large-scale network enables quantification of topological properties that might be more sensitive to the widespread alterations in ASD.

Despite growing interest in network-based approaches, previous studies have yielded inconsistent and sometimes contradictory findings. Some reported decreased network efficiency in ASD (Lewis et al., 2014; Roine et al., 2015), whereas others observed increased efficiency (Cai et al., 2022; S.-J. Li et al., 2018; Qin et al., 2018), while several found no significant group differences (He et al., 2021; Zheng et al., 2021). These inconsistencies may result from the substantial clinical heterogeneity within the autism spectrum, where different ASD subtypes may reflect unique brain systems and behavioral phenotypes (Qi et al., 2020). Treating ASD as a single, homogeneous category likely obscures meaningful neurobiological variation, contributing to these inconsistent results. Therefore, stratifying ASD into meaningful subgroups and using graph-theoretic methods may offer deeper insights into the etiology of the disorder (Hernandez et al., 2015).

Language ability is one particularly informative dimension for delineating ASD subgroups (Lombardo et al., 2015; Rapin et al., 2009; Xiao et al., 2022). Although language impairment is not a diagnostic criterion in the Diagnostic and Statistical Manual of Mental Disorders, Fifth Edition (DSM-5), it is a common and clinically relevant feature of ASD (Pickles et al., 2009; Tager-Flusberg, 2015). While several studies have examined structural brain differences associated with varying levels of language ability (Hu et al., 2025; Naigles et al., 2017; Xiao et al., 2025), few have systematically tested how language ability modulates the relationship between brain network topological organization and symptom severity across the spectrum.

The present study aimed to address these gaps by investigating the effect of language ability on the structural topology of white matter networks in children with ASD. Instead of treating ASD as a single group, we clustered our ASD sample into two subgroups based on language ability: ASD with lower language ability (ASD-LL) and ASD with higher language ability (ASD-HL). By comparing these two subgroups to typically developing (TD) children, we sought to determine whether structural network alterations represent a general feature of ASD or are primarily driven by those with more pronounced language deficits. We first characterized age-related trajectories of global network organization across groups and examined network alterations at both the global and regional levels. We hypothesized that language ability would shape white matter network characteristics, with the ASD-LL subgroup showing greater disruptions in global and local network organization as well as altered developmental trajectories. Furthermore, we examined the relationships among structural network properties, language ability, and autism-related symptoms both within ASD subgroups and across the ASD sample, and investigated whether language ability moderates the relationship between white matter network topology and symptom severity. Together, these analyses aim to clarify the contribution of language ability to the neural architecture of ASD and to provide new insights into the neurobiological basis of phenotypic heterogeneity in autism, thereby informing future intervention strategies targeting language-related neural circuits.

## 2 Methods

### 2.1 Participants

In this study, 120 participants (79 ASD/41 TD, 87 boys and 33 girls) aged 17 to 95 months were recruited and scanned at the Third Affiliated Hospital of Guangzhou Medical University, China, from August 2024 to July 2025. To ensure the groups were matched on age and gender, 6 children with ASD (age < 20 months) and 4 TD children (age > 94 months) were excluded. Following a quality check of the MRI data, an additional 7 participants (6 ASD/1 TD) were excluded due to poor data quality. The final sample for analysis consisted of 103 participants: 67 children with ASD (54 boys; mean age = 49.42 ± 19.77 months) and 36 TD children (23 boys; mean age = 59.78 ± 23.24 months).

Participants with ASD met the DSM-5 diagnostic criteria and were assessed using the Childhood Autism Rating Scale (CARS), administered by a qualified clinician. The guardians of all participants completed the Autism Behavior Checklist (ABC; Krug et al., 1980). TD children were screened by experienced pediatric clinicians to confirm the absence of language impairment and social-communication difficulties. In addition, each participant was evaluated with the Griffiths Development Scales-Chinese (GDS-C), which measures six developmental domains: Locomotor, Personal-social, Language, Eye-hand coordination, Performance, and Practical reasoning (Li et al., 2020). Since Practical reasoning is administered only to children over three years old, it was not assessed in all participants; thus, only the first five domains were analyzed in this study. The developmental quotient (DQ), calculated as (developmental age / chronological age) × 100, was used to assess developmental levels across these domains. Global DQ was computed as the arithmetic mean of the five available GDS-C domains.

All children were able to communicate in Mandarin, with normal hearing and no known family history of major neurodevelopmental or psychiatric disorders, including ASD, schizophrenia, or bipolar disorder. Family history information was obtained through structured caregiver interviews during the initial clinical assessment. All behavioral and developmental assessments were administered in Mandarin using the standardized Mandarin version of the GDS-C by trained and experienced pediatric clinicians. Prior to testing, clinicians confirmed that each child was able to understand Mandarin instructions, thereby minimizing the likelihood that limited language comprehension affected assessment performance. This study was approved by the Ethics Committee of the Third Affiliated Hospital of Guangzhou Medical University ([2025]074), and informed consent was obtained from the parents or guardians of all participants.

To account for the range of language abilities within the ASD sample, participants were divided into subgroups based on their Language DQ. Given the lack of a standard classification criterion for defining language deficits, K-means clustering was implemented using the “kmeans” function in MATLAB (R2020b). To determine the optimal number of clusters, candidate solutions ranging from 2 to 5 clusters were evaluated using the average silhouette score. The two-cluster solution yielded the highest silhouette score (0.836) and was therefore selected. Participants were subsequently classified into a higher-language subgroup (ASD-HL) and a lower-language subgroup (ASD-LL).

### 2.2 MRI data acquisition

To ensure that participants remained asleep during the MRI scan, children under 6 years of age were sedated with oral chloral hydrate (0.5 mL/kg, up to a maximum dose of 10 mL at a 0.5% concentration) administered before scanning. This protocol aligns with its widespread use in previous studies of brain morphology in young children (Bassi et al., 2011; Liu et al., 2021). All sedated children were continuously monitored by trained medical staff throughout the scan to ensure their safety.

Diffusion-weighted imaging (DWI) data were acquired using a 3.0 T GE SIGNA Architect scanner with a 48-channel head coil. The acquisition utilized a spin-echo echo-planar imaging (SE-EPI) sequence with the following parameters: repetition time (TR) = 8325 ms, echo time (TE) = 76.4 ms, flip angle = 90°, slice thickness = 2 mm. Parallel imaging (in-plane acceleration factor = 2) and multiband imaging (acceleration factor = 2) were also used. The diffusion scheme consisted of 32 directions with b-value of 1000 and one b = 0 volume, acquired with an anterior-to-posterior (A/P) phase-encoding direction. The acquisition duration was 291 s.

Structural T1-weighted (T1) scans were also acquired with the following acquisition parameters: TR = 2532 ms, TE = 2.99 ms, inversion time = 1000 ms, field of view = 240 × 240 mm^2^, flip angle = 8°, slice thickness = 1 mm, reconstruction matrix = 512 × 512. The scan duration lasted about 205 s.

### 2.3 MRI data preprocessing

Imaging data were preprocessed using the Diffusion Connectome Pipeline (DCP) (Huang et al., 2024) toolbox and SPM12 in MATLAB (R2020b). First, all DICOM data were converted to the NIfTI format using dcm2niix, and the DWI images were corrected for head motion and eddy-current distortions using the eddy tool in FSL (https://www.nitrc.org/projects/fsl/). Susceptibility distortion correction was not performed because reverse phase-encoded b0 images were not available. Then the diffusion tensor was estimated at each voxel using the ‘dti_recon’ command, from which fractional anisotropy (FA), mean diffusivity, axial diffusivity, and radial diffusivity were calculated. All raw DWI volumes and reconstructed tensor images were visually inspected, and scans with severe geometric distortion, signal dropout, or prominent artifacts were excluded.

### 2.4 Structural network construction

We constructed whole-brain structural networks for each participant using the graph-theoretical approach. Network nodes were defined as the 90 regions (45 per hemisphere) based on the Automated Anatomical Labeling (AAL) atlas (Tzourio-Mazoyer et al., 2002). Each individual's T1-weighted image was linearly co-registered to the native diffusion space (b0 image). The co-registered T1 image was then spatially normalized to the MNI space to generate a nonlinear transformation matrix, and the inverse transformation was applied to warp the AAL atlas from standard space into the individual's native diffusion space to define network nodes. Edges were defined by white matter pathways connecting pairs of nodes, reconstructed using deterministic tractography. Tracking was initiated from white matter seed points and terminated when the streamline (i) reached a region with low fractional anisotropy (FA < 0.2), (ii) turned at an angle greater than 45°, or (iii) entered gray matter.

For each participant, we generated a 90 × 90 fiber number (FN) matrix representing the number of reconstructed streamlines between each pair of regions. Two regions were considered structurally connected if at least three fibers were detected (threshold = 3), a criterion widely used in previous research (Bai et al., 2012; Li et al., 2018; Shu et al., 2011). To test the robustness of this choice, thresholds from 3 to 10 fibers were also evaluated. A binary, symmetric adjacency matrix was constructed for each participant: edges were coded as 1 if the FN exceeded the threshold (3 fibers) and 0 otherwise. These matrices were then used for network analysis performed with the GRETNA toolbox (Wang et al., 2015).

### 2.5 Network topological properties

Global network metrics were calculated following prior research (Rubinov and Sporns, 2010), including global efficiency (Eg), local efficiency (Eloc), characteristic path length (Lp), clustering coefficient (Cp), and small-world parameters (Lambda, Gamma, Sigma). Eg measures the efficiency of parallel information transfer across the network, with a higher value indicating greater network integration. Eloc reflects the efficiency within local neighborhood, indicating network segregation and fault tolerance. Small-world properties were assessed by comparing the empirical networks to 100 matched random networks generated with the Markov wiring algorithm, which preserves the degree distribution of the real network (Maslov and Sneppen, 2002). Gamma represents the normalized clustering coefficient, Lambda represents the normalized characteristic path length, and Sigma is the small-worldness index, calculated as the ratio of Gamma to Lambda (Sigma = Gamma / Lambda). A small-world network is characterized by high clustering and short path length (Sigma >1).

We also calculated three nodal metrics: degree centrality (Dc), the number of connections a given node has; nodal efficiency (Ne), the average efficiency of information transfer between a node and all other nodes in the network; and nodal shortest path length (Nlp), the average shortest path length between a node and all other nodes.

Regions were identified as hubs if their Ne was greater than 1 SD above the network mean. Following the identification of shared and group-specific hubs, we conducted supplementary analyses to characterize their system-level functional distribution. Specifically, the MNI coordinates of the identified hubs were projected onto the Yeo 7-network functional atlas (Thomas Yeo et al., 2011; 1-mm resolution) using a 6-mm neighborhood probability approach. The proportional distribution of each group's hubs across the seven functional systems was then calculated. To assess the behavioral relevance of hub topology, partial correlation analyses were performed between the Ne of group-specific hubs and clinical measures (Language DQ and ABC scores), controlling for age and gender. These analyses were conducted separately within the ASD and TD groups.

### 2.6 Age-related trajectories of network topology

To characterize age-related developmental patterns in white matter network topology, we modeled five global network metrics (Eg, Eloc, Cp, Lp, and Sigma) as a function of age (months) within each diagnostic subgroup. Given that white matter development during early childhood may follow nonlinear developmental trajectories (Kim et al., 2026), both a linear model and a three-parameter asymptotic exponential model were fitted for each metric. Model performance was evaluated using the Akaike information criterion (AIC) and adjusted R^2^, with lower AIC and higher adjusted R^2^ indicating a better fit.

To further test whether age-related developmental trajectories differed across diagnostic subgroups, regression models including group, age, and the group × age interaction were fitted for each of the five global network metrics, with gender included as a covariate. The significance of the interaction term was used to assess whether the relationship between age and network topology differed across groups.

### 2.7 Network-based-statistic approach

Group differences in structural brain connectivity were identified using the Network-Based Statistic (NBS), which controls the family-wise error rate by evaluating clusters of connected edges that exceed a primary threshold (Zalesky et al., 2010). Analyses were conducted with the NBS toolbox (v1.2) in MATLAB (R2020b). We performed three independent-sample *t*-tests (ASD-LL vs. ASD-HL; ASD-LL vs. TD; ASD-HL vs. TD), with age and gender included as nuisance covariates. For each test, statistical significance was assessed using 5000 permutations. To adjust for multiple group comparisons, Bonferroni correction was applied, resulting in a corrected threshold of *p* < 0.0167.

### 2.8 Statistical analyses

Statistical analyses were performed using SPSS (v22.0) and MATLAB (R2020b). One-way analysis of variance (ANOVA) was used to compare age, clinical measurements, and behavioral scores across groups, with Bonferroni correction applied for post hoc tests. A chi-square test was used to compare the gender distribution across the three groups.

For global network metrics, both the area under the curve (AUC) across thresholds of 3–10 fibers and values at the standard threshold (3 fibers) were analyzed. Group differences in global and nodal network metrics were assessed using analysis of covariance (ANCOVA), with age and gender included as covariates. Nodal-level comparisons were corrected for multiple comparisons using the false discovery rate (FDR).

Partial correlation analyses, controlling for age and gender, were conducted to examine the relationships between brain network metrics and clinical or behavioral data. These analyses were performed both within each ASD subgroup and across all ASD participants combined.

To further examine the relationship between network topology and disrupted structural connectivity, the mean FN (used as a proxy for connection strength) within the NBS-identified component was calculated for each subject in the ASD group. Partial correlations (controlling for age and gender) were then performed between mean FN and clinical measures (ABC scores and Language DQ) within each ASD subgroup.

To examine the functional relevance of the observed network alterations, we conducted a hypothesis-driven region-of-interest (ROI) analysis focusing on language-network regions. The language-related nodes were defined using the probabilistic language atlas (Lipkin et al., 2022), thresholded at a probability greater than 0.3. These regions were then matched to the NBS subnetwork. Partial correlations between Ne of these language-network nodes and Language DQ or ABC scores were calculated while controlling for age and gender.

### 2.9 Moderation analysis

Given the subgroup-specific brain-behavior associations, we further examined whether language ability moderated these associations independently of subgroup classification. The moderation analysis was performed using the PROCESS macro for SPSS (Model 1; Hayes, 2017), with ABC scores as the independent variable (X), brain network metrics as the dependent variable (Y), and Language DQ as the moderator (W). All variables were standardized prior to analysis, and age and gender were included as covariates. The moderation effect was evaluated by the significance of the interaction term (X × W, *p* < 0.05). Significant interactions were probed with simple slopes analyses at low (mean − 1 SD) and high (mean + 1 SD) levels of the moderator.

### 2.10 Specificity of the moderation effect

To further characterize the observed moderation effect, we conducted a series of supplementary analyses to evaluate its topological, developmental, and diagnostic specificity.

First, to examine the topological specificity, we repeated the moderation analysis using the mean FN of the NBS-identified subnetwork, rather than Eg, as the dependent variable.

Second, to examine whether the moderating effect was specific to language ability rather than reflecting broader developmental functioning, we performed parallel moderation analyses, in which the other four domains (Locomotor, Personal-social, Eye-hand coordination, and Performance) were entered individually as alternative moderators.

Finally, to assess diagnostic specificity, we examined whether the association between Language DQ and Eg differed according to diagnostic classification across the full sample (*n* = 103). Moderation analyses were performed using both a binary diagnostic model (pooled ASD vs. TD) and a three-group model (ASD-LL, ASD-HL, and TD).

### 2.11 Robustness analyses

To evaluate the robustness of the primary findings, we performed two complementary analyses.

First, to assess the stability of the language-based subgrouping, we repeated the primary group comparisons using a median split of Language DQ instead of K-means clustering.

Second, to examine whether the findings depended on the choice of network edge weighting, we reconstructed structural connectivity matrices using the mean FA of connecting streamlines as edge weights. Consistent with previous structural network studies (Niu et al., 2018), graph-theoretical metrics were calculated across a sparsity range of 0.10 to 0.35 with a step size of 0.01, and the AUC was calculated for all network measures.

## 3 Results

### 3.1 Demographic and clinical data

**Fig. 1:**
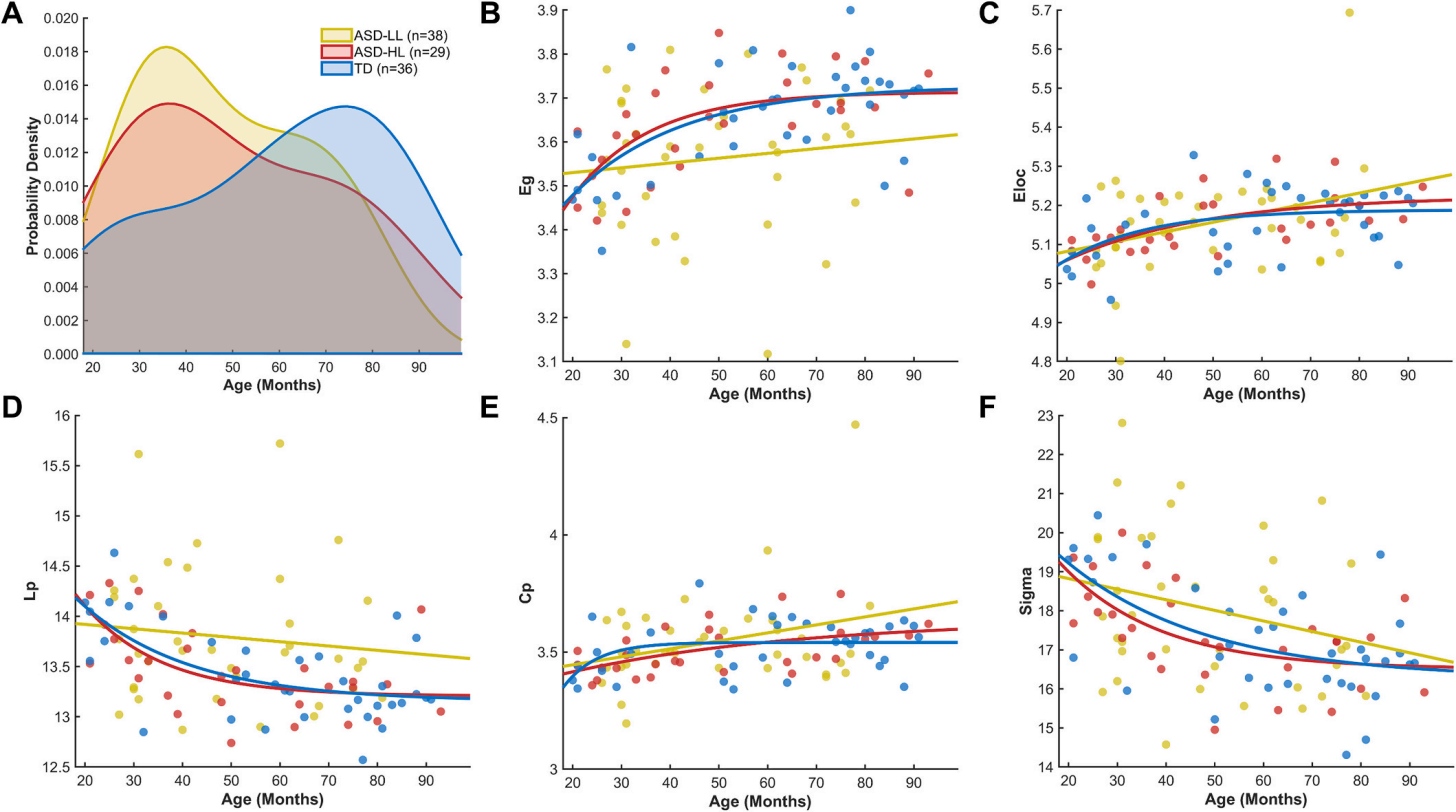
Age distribution and age-related trajectories of global network topology across diagnostic subgroups. (A) Age distribution of the three diagnostic subgroups: lower-language ASD (ASD-LL), higher-language ASD (ASD-HL), and typically developing children (TD). (B—F) Fitted age-related trajectories for five global network topology metrics: global efficiency (Eg), local efficiency (Eloc), clustering coefficient (Cp), characteristic path length (Lp), and small-worldness (Sigma). Each point represents an individual participant, and the fitted lines/curves depict the age-related trajectories estimated by the exponential or linear models for each subgroup.

K-means clustering identified two distinct ASD subgroups: Cluster 1 (*n* = 38, 30 boys/8 girls) with a centroid of 29.8 (ASD-LL) and Cluster 2 (*n* = 29, 24 boys/5 girls) with a centroid of 62.4 (ASD-HL). As shown in Table 1, the three groups did not differ significantly in age (*F*_(2,100)_ = 2.88, *p* = 0.06; Fig. 1A) or gender (*p* = 0.17). However, significant group differences were observed in ABC, CARS, and all five domains of the GDS-C (*p*s < 0.001).

**Table 1:** Demographic, clinical, and behavioral characteristics of three groups.

Variables	Group (M ± SD)			ANOVA	
	ASD-LL	ASD-HL	TD	*F*	*p*
Age	48.63 ± 18	50.45 ± 22.16	59.78 ± 23.24	2.88	0.06
Gender	30 M/8F	24 M/5F	33 M/13F	3.59 ^b^	0.17
ABC ^a^	52.05 ± 22.58	47.83 ± 20.76	12.12 ± 10	47.23	<0.001
CARS ^a^	36.15 ± 5.92	31.26 ± 5.24	13.67 ± 4.32	177.19	<0.001
GDS-C					
*(A) Locomotor*	61.42 ± 15.84	82.73 ± 12.84	97.87 ± 11.63	66.62	<0.001
*(B) Personal-social*	40.89 ± 12.49	64.54 ± 11.17	95.98 ± 7.4	251.82	<0.001
*(C) Language*	29.82 ± 9.15	62.39 ± 11.01	97.4 ± 12.98	340.63	<0.001
*(D) Eye-hand coordination*	48.6 ± 15.61	71.98 ± 17.51	93.32 ± 8.34	92.42	<0.001
*(E) Performance*	55.42 ± 19.04	77.61 ± 17.42	96.25 ± 7.98	64.08	<0.001

Notes: ASD-LL, autism spectrum disorder with lower language ability; ASD-HL, autism spectrum disorder with higher language ability; TD, typically developing; GDS-C, the Griffiths Development Scales-Chinese; M, mean; SD, standard deviation; ANOVA, analysis of variance. ^a^ ABC and CARS scores were available for 37 participants in the ASD-LL subgroup, 28 in the ASD-HL subgroup, and 34 in the TD group. ^b^ The chi-square value.

Post hoc tests with Bonferroni correction indicated that both ASD subgroups scored significantly higher than the TD group on the ABC and CARS (*p*s < 0.001). While the two ASD subgroups did not differ on ABC scores (*t*_(64)_ = 0.91, *p* > 0.05), the ASD-LL subgroup showed significantly higher CARS scores than the ASD-HL subgroup (*t*_(64)_ = 3.78, *p* < 0.001). For all five GDS-C domains, scores followed the same order: TD > ASD-HL > ASD-LL (all pairwise *p*s < 0.001).

### 3.2 Developmental trajectories of global network topology across groups

To examine age-related patterns in white matter network topology, linear and asymptotic exponential models were fitted for five global network metrics (Eg, Eloc, Cp, Lp, and Sigma) within each diagnostic subgroup. Fig. 1B-F illustrates the best-fitting age-related trajectory for each metric and subgroup. Model comparison using AIC and adjusted R^2^ indicated that, for the ASD-HL and TD groups, the asymptotic exponential model provided a better fit than the linear model for Eg and Lp (Table S1). In contrast, the linear model was generally favored for the ASD-LL subgroup across the five global network metrics (Table S1).

No significant group × age interactions were observed for any metric (all *ps* > 0.05), suggesting that although the fitted trajectories differed descriptively among groups, our cross-sectional data did not provide statistical evidence for different developmental slopes.

### 3.3 Alterations in global network properties

**Fig. 2:**
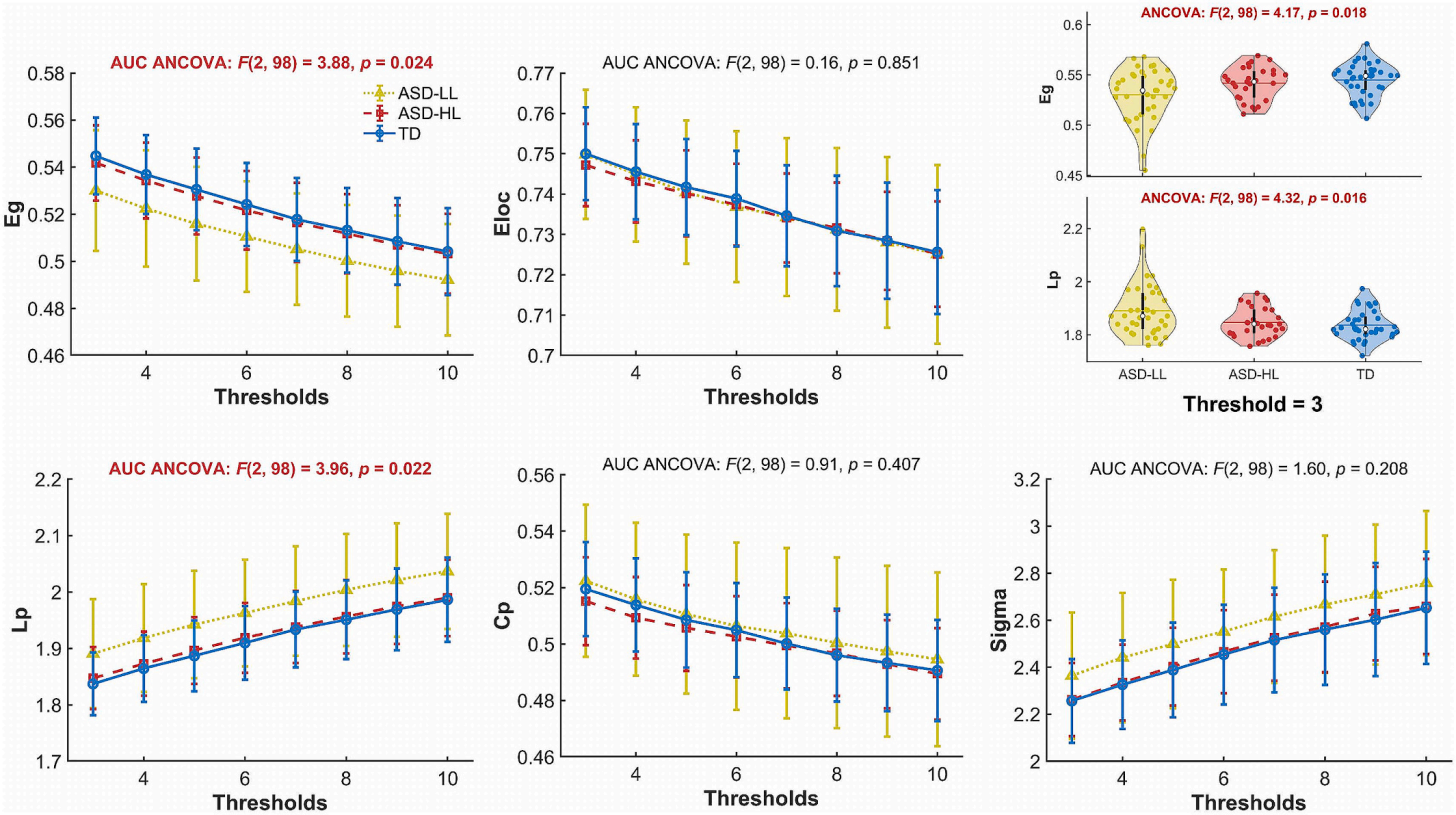
Group comparisons of global network properties. Line plots show five global network metrics for each group across a range of thresholds (defining an edge by a minimum of 3 to 10 fibers). The ANCOVA analyses on the area under the curve (AUC) found significant group differences for global efficiency (Eg) and characteristic path length (Lp), but not for local efficiency (Eloc), the clustering coefficient (Cp), or the small-world index (Sigma). Violin plots are presented to visualize the distributions for only the two significant metrics (Eg and Lp) at a representative threshold of 3 fibers. Abbreviations: ASD-LL, autism spectrum disorder with lower language ability; ASD-HL, autism spectrum disorder with higher language ability; TD, typically developing.

We compared global network properties among the three groups using ANCOVA, including Eg, Eloc, and small-world parameters. As shown in Fig. 2, AUC analyses revealed a significant group effect on Eg (*F*_(2,98)_ = 3.88, *p* = 0.02), but not on Eloc (*F*_(2,98)_ = 0.16, *p* = 0.85). All groups exhibited a small-world topology (Sigma >1). There were significant group differences in Lp (*F*_(2,98)_ = 3.97, *p* = 0.02) and Lambda (*F*_(2,98)_ = 3.66, *p* = 0.03), but not in Cp (*F*_(2,98)_ = 0.91, *p* = 0.41) or Gamma (*F*_(2,98)_ = 1.95, *p* = 0.15).

As patterns were consistent across thresholds (3–10 fibers), we reported results at the representative threshold of 3 fibers. At this threshold, group effects remained significant for Eg (*F*_(2,98)_ = 4.17, *p* = 0.018) and Lp (*F*_(2,98)_ = 4.32, *p* = 0.016) (Fig. 2). Post hoc tests revealed that children in the ASD-LL subgroup showed significantly decreased Eg (*t*_(98)_ = −2.59, *p* = 0.03) and increased Lp (*t*_(98)_ = 2.61, *p* = 0.028) compared to the TD group. Differences between the ASD-LL and ASD-HL subgroups were marginally significant in Eg (*t*_(98)_ = −2.30, *p* = 0.06) and Lp (*t*_(98)_ = 2.37, *p* = 0.052). No significant differences were found between the ASD-HL subgroup and the TD group (*p*s > 0.05).

### 3.4 Nodal characteristics, hub identification, and hub-behavior associations

We next examined nodal network characteristics, including Ne, Dc, and Nlp. Although several nodes showed group differences at the uncorrected level, none remained significant after FDR correction (all corrected *p*s > 0.05).

**Fig. 3:**
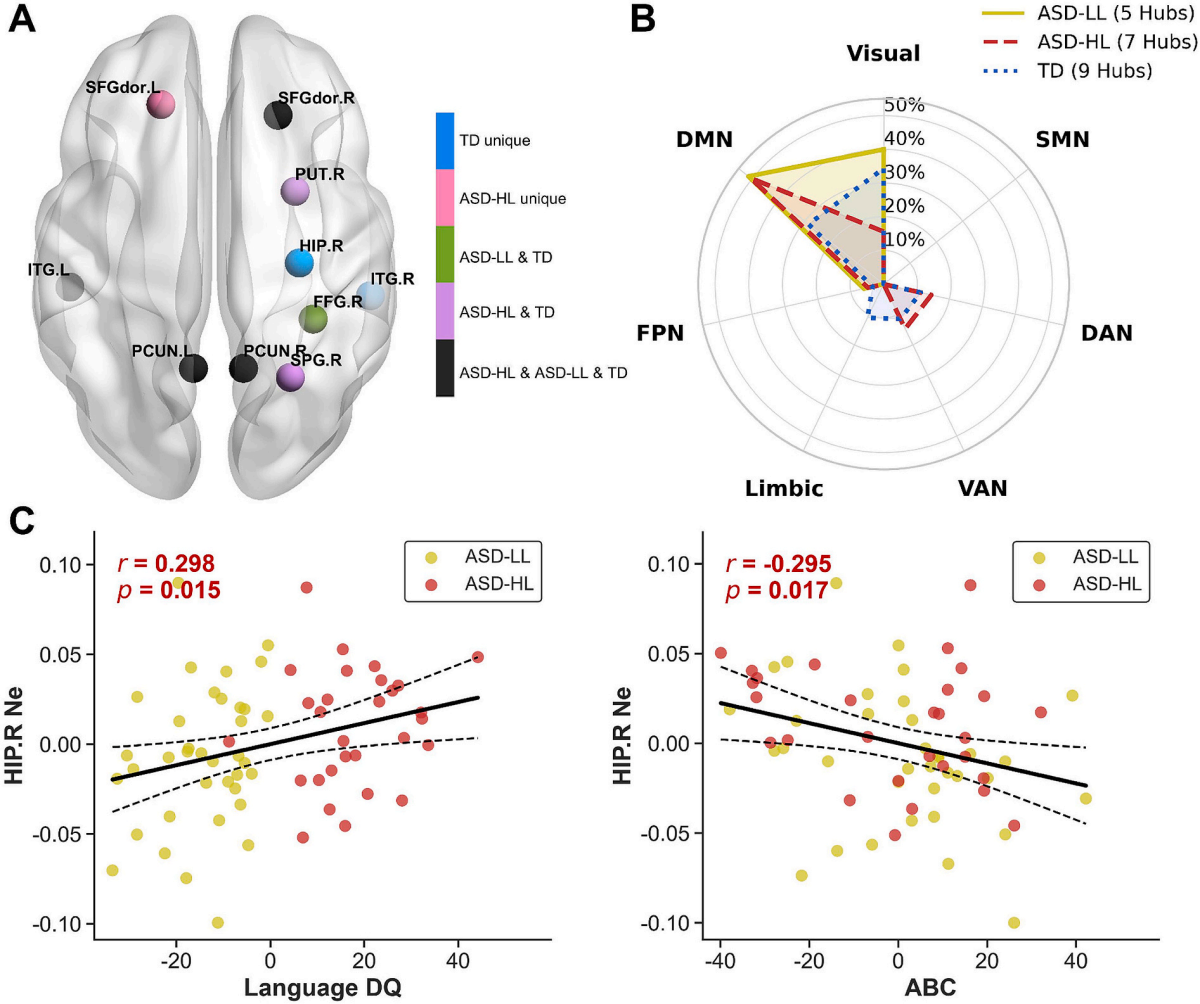
Structural network hubs, their functional network mapping, and hub-behavior associations. (A) Spatial visualization of shared and group-specific structural network hubs. Hubs were defined as nodes with group-average nodal efficiency (Ne) greater than one standard deviation above the network mean. (B) Functional distribution of hubs mapped onto the Yeo 7-network atlas. The radar plot demonstrates the proportional distribution of each group's hubs across Visual Network, Somatomotor Network (SMN), Dorsal Attention Network (DAN), Ventral Attention Network (VAN), Limbic Network, Frontoparietal Network (FPN), and Default Mode Network (DMN). (C) Partial correlation analyses (controlling for age and gender) show that Ne of the TD-unique right hippocampus (HIP.R) is significantly associated with Language DQ and autism symptom severity (ABC scores) in the ASD group. No significant associations were observed in TD children. *Note*: statistical significance is reported at the uncorrected level. The single data point with a negative Language DQ residual in the ASD-HL group reflected the age- and gender-adjustment procedure. Sensitivity analyses confirmed that reclassifying this case into the ASD-LL group did not alter the statistical significance of any network topological metrics or correlation analyses.

Regarding hub identification, four regions were consistently identified as hubs across all groups (Fig. 3A, Table S2): the right dorsal superior frontal gyrus (SFGdor.R), the bilateral precuneus (PCUN.L, PCUN.R), and the left inferior temporal gyrus (ITG.L). The ASD-HL and TD groups shared two additional hubs in the right superior parietal gyrus (SPG.R) and right putamen (PUT.R), whereas these hubs were absent in the ASD-LL subgroup. The ASD-LL and TD groups shared one additional hub in the right fusiform gyrus (FFG.R). In addition, the TD group exhibited two unique hubs located in the right hippocampus (HIP.R) and right inferior temporal gyrus (ITG.R). No unique hubs were identified in the ASD-LL subgroup.

To characterize the system-level functional organization of these hubs, they were projected onto the Yeo 7-network functional atlas (Fig. 3B). Hubs in both ASD subgroups were predominantly distributed within the default mode network (DMN), whereas the TD group exhibited a broader distribution across multiple functional systems, including the DMN, visual, limbic, ventral attention, and dorsal attention networks.

Partial correlation analyses controlling for age and gender showed that, within the ASD group, higher Ne of the TD-specific hub (i.e., HIP.R) was significantly associated with higher Language DQ (*r* = 0.298, *p* = 0.015) and lower ABC scores (*r* = −0.295, *p* = 0.017) (Fig. 3C). In the TD group, however, no significant associations were found (all *p*s > 0.05).

### 3.5 NBS analysis revealed a decreased subnetwork in the ASD-LL subgroup

**Fig. 4:**
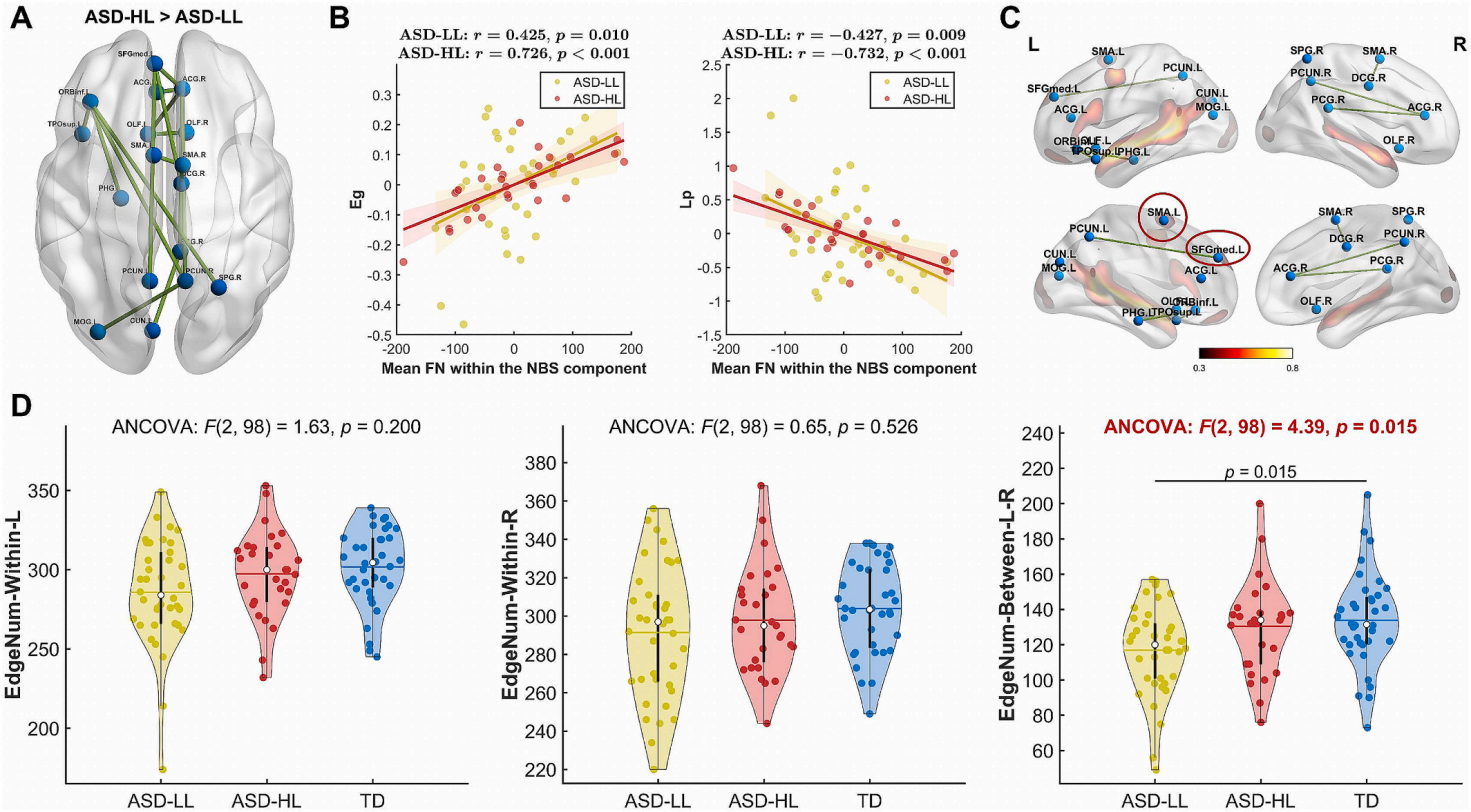
The subnetwork identified by the network-based statistic (NBS) and the validation of modular interaction analyses. (A) The subnetwork consisted of 17 nodes and 16 edges, with all edges showing decreased connectivity in children with ASD-LL compared to the ASD-HL group. Nodes and connections are mapped onto cortical surfaces using the BrainNet Viewer (https://www.nitrc.org/projects/bnv/). (B) Partial correlations between the mean connection strength (mean FN) of the NBS-identified subnetwork and global efficiency (Eg) and shortest path length (Lp) within the ASD-HL and ASD-LL subgroups. (C) Overlap between the NBS-identified subnetwork and the predefined probabilistic language network. Only two language-related regions overlapped with the NBS component: the left supplementary motor area (SMA.L) and the left medial superior frontal gyrus (SFGmed.L). (D) Group comparisons of intra- and inter-hemispheric structural connectivity. Violin plots illustrate the total fiber number (FN) within the left hemisphere (left), within the right hemisphere (middle), and between the two hemispheres (right) for the ASD-LL, ASD-HL, and TD groups. A significant group difference was found for inter-hemispheric connectivity, with lower inter-hemispheric FN in the ASD-LL subgroup than in the TD group. Abbreviations: L, left hemisphere; R, right hemisphere; ASD-LL, autism spectrum disorder with lower language ability; ASD-HL, autism spectrum disorder with higher language ability; TD, typically developing.

NBS analysis identified one subnetwork with significantly decreased connectivity in the ASD-LL subgroup compared to the ASD-HL subgroup (*p* = 0.014, 5000 permutations). As illustrated in Fig. 4A and Table 2, this subnetwork comprised 17 nodes and 16 edges, including 10 inter-hemispheric and 6 intra-hemispheric connections (3 within each hemisphere). The right anterior cingulate gyrus (ACG) emerged as the most central node (degree = 5), followed by the left inferior orbital frontal gyrus (ORBinf; degree = 4), and the left medial superior frontal gyrus (SFGmed) and right precuneus (degree = 3 each). No significant subnetworks were observed in other group comparisons (i.e., ASD-HL vs. TD, ASD-LL vs. TD; *p*s > 0.05).

**Table 2:** Subnetwork with decreased connections in the ASD-LL subgroup.

Edges	Region A				Region B			
	Full name	Coordinates (MNI)			Full name	Coordinates (MNI)		
		x	y	z		x	y	z
**Intra-hemisphere**								
SFGmed.L - PCUN.L	left medial/superior frontal	−4.8	49.17	30.89	left precuneus	−7.24	−56.07	48.01
ORBinf.L - TPOsup.L	left inferior orbital part	−35.98	30.71	−12.11	left superior temporal pole	−39.88	15.14	−20.18
ORBinf.L - PHG.L	left inferior orbital part	−35.98	30.71	−12.11	left parahippocampus	−21.17	−15.95	−20.7
SMA.R - DCG.R	right supplementary motor area	8.62	0.17	61.85	right middle cingulum	8.02	−8.83	39.79
ACG.R - PCG.R	right anterior cingulum	8.46	37.01	15.84	right posterior cingulum	7.44	−41.81	21.87
ACG.R - PCUN.R	right anterior cingulum	8.46	37.01	15.84	right precuneus	9.98	−56.05	43.77
**Inter-hemisphere**								
SFGmed.L - ACG.R	left medial/superior frontal	−4.8	49.17	30.89	right anterior cingulum	8.46	37.01	15.84
SFGmed.L - DCG.R	left medial/superior frontal	−4.8	49.17	30.89	right middle cingulum	8.02	−8.83	39.79
SMA.L - SMA.R	left supplementary motor area	−5.32	4.85	61.38	right supplementary motor area	8.62	0.17	61.85
MOG.L - PCUN.R	left middle occipital	−32.39	−80.73	16.11	right precuneus	9.98	−56.05	43.77
CUN.L - PCG.R	left cuneus	−5.93	−80.13	27.22	right posterior cingulum	7.44	−41.81	21.87
ACG.L - ACG.R	left anterior cingulum	−4.04	35.4	13.95	right anterior cingulum	8.46	37.01	15.84
ORBinf.L - PCUN.R	left inferior orbital part	−35.98	30.71	−12.11	right precuneus	9.98	−56.05	43.77
ORBinf.L - SPG.R	left inferior orbital part	−35.98	30.71	−12.11	right superior parietal	26.11	−59.18	62.06
OLF.L - ACG.R	left olfactory	−8.06	15.05	−11.46	right anterior cingulum	8.46	37.01	15.84
OLF.L - OLF.R	left olfactory	−8.06	15.05	−11.46	right olfactory	10.43	15.91	−11.26

Notes: Connection was defined as the number of fiber tracts (edge) connecting any two given nodes. ASD-LL, autism spectrum disorder with lower language ability.

### 3.6 Association of NBS subnetwork strength with global network metrics and language-related nodes

In both ASD subgroups, mean FN within the NBS-identified subnetwork was significantly associated with global network topology (Fig. 4B). For the ASD-LL group, mean FN was positively associated with Eg (*r* = 0.425, *p* = 0.01) and negatively with Lp (*r* = −0.427, *p* = 0.01), accounting for 18.09% and 18.22% of the residual variance after adjusting for age and gender, respectively. Stronger associations were observed in the ASD-HL subgroup (Eg: *r* = 0.726, *p* < 0.001, 52.75% variance explained; Lp: *r* = −0.732, *p* < 0.001, 53.52% variance explained).

In contrast, mean FN of the NBS-identified subnetwork was not significantly associated with clinical autism severity (ABC scores) or language ability (Language DQ) in either ASD subgroup (all *ps* > 0.05).

To determine whether the NBS-identified subnetwork involved language-related regions, the subnetwork showing reduced connectivity in the ASD-LL group (ASD-HL > ASD-LL) was mapped onto a predefined probabilistic language network (Fig. 4C). Only two language-related nodes overlapped with the NBS component: the left supplementary motor area (SMA.L) and the left medial superior frontal gyrus (SFGmed.L). Partial correlation analyses further revealed that Ne of neither region was significantly associated with Language DQ or ABC scores in either ASD subgroup (all *p*s > 0.05).

### 3.7 Decreased inter-hemispheric connections in the ASD-LL subgroup

To further investigate the inter-hemispheric effects, we compared intra- and inter-hemispheric connections by defining left- and right-hemisphere modules (45 nodes each).

As shown in Fig. 4D, no significant group differences were found in the intra-hemispheric connections (Within-L: *F*_(2,98)_ = 1.63, *p* = 0.20; Within-R: *F*_(2,98)_ = 0.65, *p* = 0.53), but inter-hemispheric connectivity differed significantly across groups (Between-L-R: *F*_(2,98)_ = 4.39, *p* = 0.015). Post hoc comparisons revealed that the ASD-LL subgroup exhibited significantly fewer inter-hemispheric connections than the TD group (*t*_(98)_ = −2.83, *p* = 0.015), but did not differ significantly from the ASD-HL group (*t*_(98)_ = −2.04, unadjusted *p* = 0.045, adjusted *p* = 0.11). No significant difference was observed between the ASD-HL group and TD group (*t*_(98)_ = −0.71, *p* = 0.76).

### 3.8 Associations between clinical data and global network characteristics in ASD subgroups

We examined the relationships between global network characteristics and clinical data (ABC scores) using partial correlation analyses, controlling for age and gender. Because Eg and Lp represent conceptually similar aspects of network integration, only Eg was included in the analyses.

**Fig. 5:**
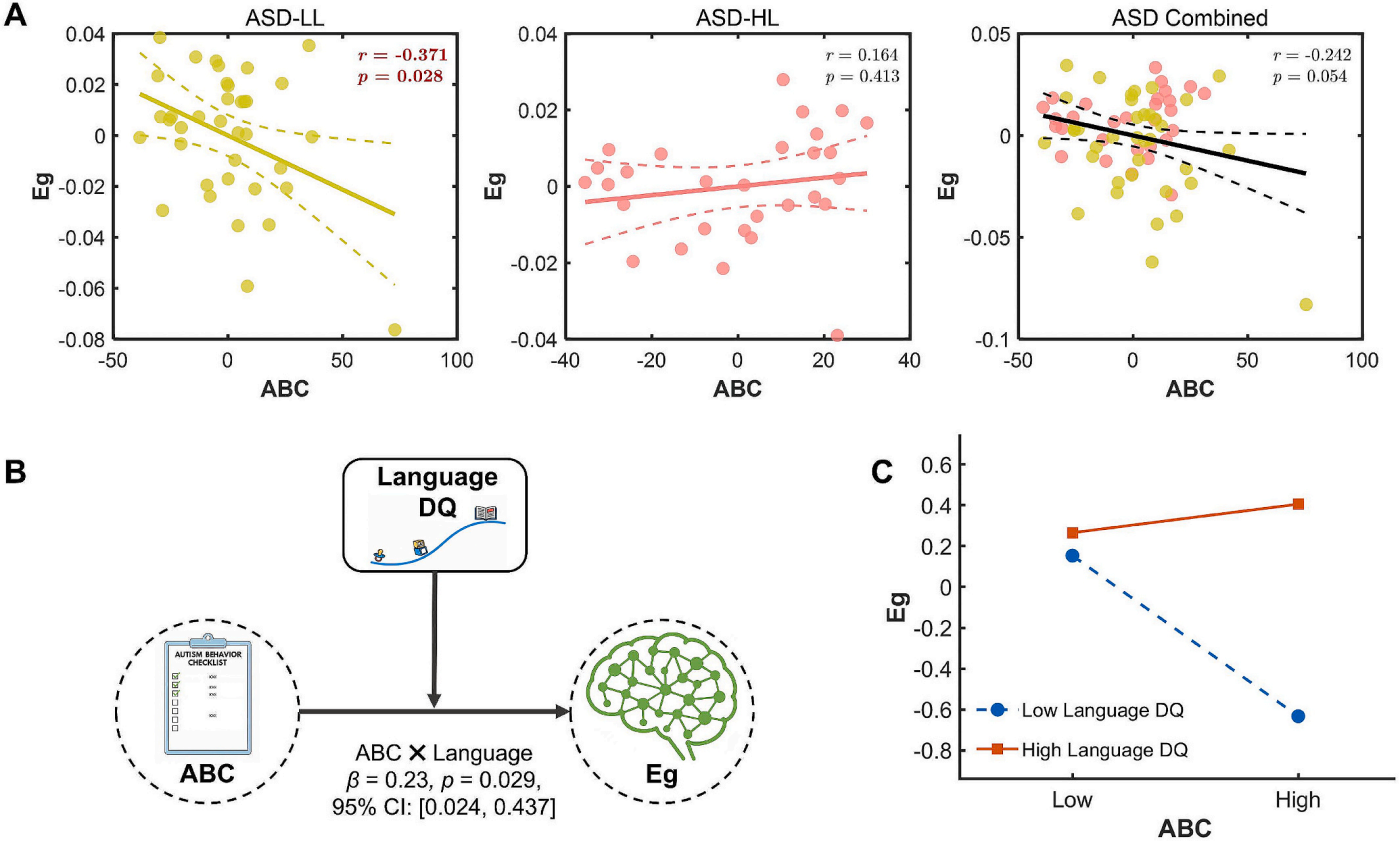
Correlation and moderation analyses between global network index, clinical symptoms, and behavioral measures. (A) The scatter plots show the relationship between global efficiency (Eg) and autism symptom severity (indexed by ABC scores) in ASD subgroups (ASD-LL and ASD-HL) and in the combined ASD sample. All partial correlations were performed with age and gender controlled. The dashed lines represent the 95% confidence interval. (B) The diagram of a moderation model illustrates how language level moderates the relationship between autism symptom severity (ABC scores) and global efficiency (Eg) in the ASD group. (C) The simple slope plot shows the conditional effect of ABC on Eg at high (mean + 1 SD) and low (mean – 1 SD) levels of Language DQ.

In the ASD-LL subgroup, Eg was significantly negatively correlated with ABC scores (*r*_(33)_ = −0.37, *p* = 0.028), but this relationship was not significant in the ASD-HL subgroup or for all ASD participants combined (*p*s > 0.05, Fig. 5A).

### 3.9 Moderating role of language ability

Given that the correlation between Eg and ABC scores was significant only in the ASD-LL subgroup but not in the ASD-HL subgroup, we next examined whether language ability continuously moderated this relationship across the entire ASD cohort, rather than acting simply as a categorical subgroup effect. A moderation analysis was therefore performed in the combined ASD sample, with Language DQ as a continuous moderator of the relationship between network efficiency (Eg) and autism symptom severity (ABC scores).

As shown in Table 3 and Fig. 5B, Language DQ significantly moderated the relationship between ABC scores and Eg (*β* = 0.23, SE = 0.10, *p* = 0.029, 95% CI [0.024, 0.437]). Simple slopes analysis showed that among children with lower language ability (1 SD below the mean), higher ABC scores were significantly associated with lower Eg (*β* = −0.39, SE = 0.15, *p* = 0.014, 95% CI [−0.70, −0.08]). In contrast, for children with higher language ability (1 SD above the mean), this relationship was non-significant (*β* = 0.07, SE = 0.16, *p* = 0.662, 95% CI [−0.25, 0.39]).

**Table 3:** Moderation analysis in regression models predicting global efficiency (Eg).

Predictor	*Standardized Coefficient (β)*	*Standard Error (SE)*	*t value*	*p value*	*F*	*R Square*
(Constant)					3.01	0.17
Age	0.19	0.12	1.59	0.12		
Gender	0.04	0.12	0.34	0.74		
ABC	−0.17	0.12	−1.39	0.17		
Language DQ	0.27	0.12	2.2	0.032		
(Constant)					3.57	0.23
Age	0.21	0.12	1.8	0.08		
Gender	0.01	0.12	0.1	0.92		
ABC	−0.16	0.12	−1.39	0.17		
Language DQ	0.29	0.12	2.43	0.02		
ABC × Language DQ	0.23	0.1	2.23	0.029		

Abbreviations: ABC, Autism Behavior Checklist; DQ, developmental quotient.

### 3.10 Specificity of the language moderation effect

To further evaluate the specificity of the observed language moderation effect, we examined its topological, developmental, and diagnostic specificity.

First, when mean FN of the NBS-identified subnetwork was used as the dependent variable, the interaction between Language DQ and ABC scores was not significant (*β* = 0.076, *p* = 0.463, 95% CI [−0.13,0.28]), suggesting that the moderating effect is specific to global network integration rather than localized subnetwork connectivity.

Second, as shown in Table 4, none of the other four GDS-C domains showed a significant moderating effect on the association between ABC scores and Eg (Performance DQ: *p* = 0.155; Eye-hand coordination DQ: *p* = 0.406; Personal-social DQ: *p* = 0.697; Locomotor DQ: *p* = 0.775). Likewise, when the estimated Global DQ was used as the moderator, the interaction term was not significant (*β* = 0.149, *t* = 1.240, *p* = 0.218).

**Table 4:** Moderation effects of GDS-C domains on the relationship between ABC scores and Eg.

Moderator (W)	Standardized Coefficient (β)	Standard Error (SE)	95% CI	t value	p value
Performance DQ	0.173	0.109	[−0.067, 0.413]	1.442	0.155
Eye-hand coordination DQ	0.103	0.126	[−0.144, 0.350]	0.836	0.406
Personal-social DQ	0.05	0.129	[−0.203, 0.303]	0.392	0.697
Locomotor DQ	0.039	0.138	[−0.233, 0.310]	0.287	0.775

Finally, the interaction between Language DQ and diagnosis was not statistically significant (*β* = 0.750, 95% CI [−0.063, 1.563], *t* = 1.831, *p* = 0.070) in the binary diagnostic model (n = 103; pooled ASD vs. TD). However, when diagnostic classification was modeled using the three phenotypic subgroups (ASD-LL, ASD-HL, and TD), the Language DQ × subgroup interaction was significant for ASD-LL relative to TD (*β* = 1.548, 95% CI [0.316, 2.780], *t* = 2.495, *p* = 0.014), but not for ASD-HL relative to TD (*β* = 0.148, 95% CI [−1.044, 1.339], *t* = 0.246, *p* = 0.806).

### 3.11 Results of robustness analyses

First, we repeated the group comparisons using a median-split of Language DQ (ASD-LL: *n* = 34; ASD-HL: *n* = 33), and observed a pattern of results comparable to the K-means-based subgrouping (Table S3).

Second, reconstructing structural networks using FA-weighted connectivity yielded group differences and brain–behavior associations that were highly consistent with the primary FN-weighted findings (Figs. S1 and S2; Table S4). The moderating effect of Language DQ on the association between ABC scores and Eg also remained significant (*β* = 0.246, SE = 0.099, *t* = 2.477, *p* = 0.016, 95% CI [0.047, 0.445]).

## 4 Discussion

In this study, we examined the effect of language deficit on brain white matter topological organization by comparing the structural network characteristics across ASD children with varying language abilities to their age- and gender-matched TD peers. Rather than representing a universal feature of ASD, reduced global network integration was confined to children with more profound developmental impairment characterized by low language ability. Furthermore, language ability moderated the association between structural network integration and autism symptom severity, suggesting that language phenotype captures a biologically meaningful dimension of ASD heterogeneity. Together, these findings highlight the value of language-informed stratification for understanding individual differences in the neurobiology of ASD.

Our study revealed that the ASD-LL subgroup exhibited disrupted global network topology, characterized by decreased global efficiency and increased characteristic path length, whereas the ASD-HL subgroup showed brain network metrics largely comparable to those of the TD group. These findings are consistent with previous structural and functional imaging research in ASD (Lewis et al., 2014; Li et al., 2021; Roine et al., 2015), and align well with the long-range underconnectivity theory of autism (Just, 2004; Khan et al., 2013; Müller et al., 2011), supporting an intrinsic deficit in long-range, parallel information processing in the brains of individuals with ASD. Importantly, our findings extend this framework by demonstrating that such global network inefficiency appears specific to the ASD subgroup with more severe language impairments, rather than being a universal feature of ASD. This language-informed stratification may also help explain inconsistencies across previous graph-theoretical studies, some of which reported increased global efficiency or no group differences (Cai et al., 2022; Li et al., 2018; Rudie et al., 2013). Because most previous studies treated ASD as a homogeneous diagnostic category, biologically meaningful subgroup differences may have been obscured. Although considerable variability remained within the ASD-LL subgroup, suggesting that language ability captures an important, but not exhaustive, dimension of ASD heterogeneity (Silleresi et al., 2020), our findings nevertheless indicate that language phenotype provides clinically and biologically informative stratification beyond diagnostic status alone.

Because the present cohort spans a period of rapid white matter maturation (Stephens et al., 2020), we further explored age-related trajectories of global network topology. Although exploratory, these findings suggest that age-related organization of white matter topology was more evident in TD and ASD-HL children than in ASD-LL children. This observation raises the possibility that children with ASD and poorer language abilities may exhibit altered or delayed maturation of large-scale structural network organization. Given the cross-sectional nature of the present study, these trajectory differences should be interpreted cautiously. Because white matter development during early childhood is known to follow nonlinear trajectories (Feng et al., 2023; Zhao et al., 2026), longitudinal studies covering broader developmental periods will be necessary to determine whether these findings reflect delayed maturation, altered developmental timing, or persistent disruption of structural network organization.

Our regional analyses provide further insight into the neurobiological basis of these global network alterations. Compared with both ASD-HL and TD children, the ASD-LL subgroup showed marked reductions in inter-hemispheric fiber connections and fewer hubs in the right hemisphere, indicating that impaired communication between distributed brain regions may underlie the observed reductions in global efficiency. In contrast, TD children showed a broader and more balanced hub distribution across multiple functional systems, whereas hubs in both ASD subgroups were more heavily concentrated within the DMN. The larger and more distributed hub profile observed in TD children may reflect a more mature and balanced white-matter network architecture (Zhao et al., 2026). Furthermore, the dissociation showing that the efficiency of language-related nodes was unrelated to autism symptom severity (ABC scores) suggests that topological alterations within the language network specifically contribute to language deficit, whereas core autistic traits may stem from broader, trans-network integration failures. Nevertheless, given that these regional analyses were ROI-driven and uncorrected for multiple comparisons, these findings must be interpreted with caution and warrant validation in future studies.

Interestingly, the connectivity differences identified by network-based statistics were not confined to the canonical language network. Instead, the altered subnetwork primarily involved distributed regions spanning the DMN and executive control network, systems that support social cognition, cognitive flexibility, executive control, and adaptive behavior (Andrews-Hanna et al., 2010; Buckner et al., 2008; Lin et al., 2019; May and Kana, 2020). Moreover, only two nodes within this subnetwork overlapped with the predefined language network, and neither showed significant associations with language ability or autism symptom severity. Together, these findings suggest that language impairment in ASD is unlikely to arise from isolated disruption of classical language regions. Rather, language ability appears to reflect broader alterations in distributed neurodevelopmental systems supporting multiple higher-order cognitive functions (Forkel and Hagoort, 2024; Roger et al., 2022). Consequently, the ASD-LL subgroup should be interpreted as a more profoundly affected neurodevelopmental phenotype rather than as a group with an isolated language deficit. Future studies with larger samples and multidimensional phenotyping are needed to identify additional subtypes within the low-language ASD population.

A particularly important finding of this study is that language ability moderated the relationship between autism symptom severity and global network integration. Specifically, greater autism symptom severity was associated with lower global efficiency only among children with poorer language abilities, whereas this relationship was absent in children with relatively preserved language development. Furthermore, this moderating effect remained specific to language ability: neither the other GDS-C developmental domains nor the estimated Global DQ significantly moderated the relationship between autism symptoms and global efficiency. These findings suggest that language may contribute unique explanatory value beyond general developmental functioning. Importantly, this moderation effect was observed only for global efficiency and not for the connectivity strength of the NBS-derived subnetwork, indicating that language primarily influences the relationship between autism symptoms and large-scale network integration rather than localized structural connectivity. Overall, these findings suggest that language ability shapes how distributed structural brain organization translates into behavioral manifestations of ASD, rather than simply reflecting overall developmental delay.

Taken together, our findings also have several important clinical implications. Language ability has traditionally been regarded as a dimension of clinical characterization in ASD (Hu et al., 2025; Xiao et al., 2025). Our results suggest that it may also serve as a biologically informative stratification marker for identifying neurodevelopmentally distinct subgroups with different patterns of structural brain organization. Incorporating language phenotype into participant stratification may therefore improve the biological characterization of ASD heterogeneity and facilitate more targeted investigation of developmental mechanisms (Duan et al., 2024; Latrèche et al., 2024). Future longitudinal and intervention studies are needed to test whether improvements in language function are accompanied by adaptive reorganization of structural brain networks and whether such changes contribute to broader improvements in cognitive and social functioning in ASD.

Several limitations of this study should be noted. First, to obtain high-quality brain images in young children, particularly those with ASD, sedation with chloral hydrate was used in children under 6 years of age. Although previous studies suggest that sedation has minimal influence on structural MRI and diffusion metrics (Forkert et al., 2016), its potential effects cannot be completely excluded. Future studies employing natural sleep or awake MRI protocols will be valuable for confirming the present findings. Second, the structural brain network in this study was constructed based on streamline count, a metric previously shown to be sensitive to ASD-related alterations (Li et al., 2018; Qian et al., 2021). Although we also replicated the main findings using FA-weighted networks, other diffusion-based metrics, such as radial diffusivity and mean diffusivity, were not examined. In addition, susceptibility distortion correction could not be performed because reverse phase-encoded images were unavailable. While strict visual quality control was used to exclude scans with prominent artifacts, future studies incorporating advanced diffusion acquisition and preprocessing methods such as Synb0-DisCo (Schilling et al., 2019), will further strengthen the robustness of diffusion-derived connectivity findings. Third, deterministic tractography may underestimate long-range connections or crossing-fiber pathways despite its high specificity (Bastiani et al., 2012). Validation using complementary tractography approaches would therefore be informative. Finally, the relatively narrow age range (2–8 years), cross-sectional design, and single-center cohort limit conclusions regarding developmental trajectories and generalizability. Larger longitudinal multi-site studies with more comprehensive neurodevelopmental phenotyping will be essential for further elucidating the biological heterogeneity of ASD. Future studies may also benefit from incorporating more detailed characterization of children's language environments to further clarify how linguistic experience contributes to developmental heterogeneity in ASD.

## 5 Conclusion

Using DTI data and graph theory analyses, we found significant alterations in global network topology and inter-hemispheric connectivity that were specific to the ASD-LL subgroup, compared to the TD group. These findings suggest that language impairment may be a key factor associated with brain structural alterations in children with ASD. Furthermore, the moderating effect of language ability on the relationship between autism symptom severity and global brain efficiency corroborates this view, highlighting the pivotal role of language in shaping brain-behavior associations in ASD. Together, these results underscore the importance of incorporating language ability as a stratification and intervention target in clinical and research settings, emphasizing the potential of language-focused therapies to promote adaptive brain network development.

## CRediT authorship contribution statement

**Conghui Su:** Writing – review & editing, Writing – original draft, Visualization, Validation, Software, Methodology, Formal analysis, Data curation. **Zhaoran Liu:** Writing – review & editing, Resources. **Shuiqun Zhang:** Writing – review & editing, Resources. **Aiwen Yi:** Writing – review & editing, Resources, Investigation. **Yaqiong Xiao:** Writing – review & editing, Supervision, Project administration, Funding acquisition, Conceptualization.

## Funding

This study was supported by the 10.13039/501100001809National Natural Science Foundation of China (32200808).

## Declaration of competing interest

The authors declare no conflict of interests.

## Data Availability

The tidy data used for the analyses reported in this article are available at https://github.com/Yaqiongxiao/asd.DTI.network.
