# Supplementary material for: Altered white matter network topology is associated with more profound language impairment in children with autism spectrum disorder
Source: Neuroimage Clin. 2026 Jul 27;51:104042. doi: 10.1016/j.nicl.2026.104042 (PMC13452417; doi:10.1016/j.nicl.2026.104042)
Supplement: Supplementary file 1 [file mmc1.docx]

**Altered White Matter Network Topology Is Associated with More Profound Language Impairment in Children with Autism Spectrum Disorder**

# **Supplementary Information**

**Table S1.** Comparisons between linear and exponential trajectories against age across diagnosis subgroups

| **Metric** | **Group** | **Linear AIC** | **Linear AdjR2** | **AsympExp AIC** | **AsympExp AdjR2** | **Delta AIC** | **Best Model** |
| --- | --- | --- | --- | --- | --- | --- | --- |
| Eg | ASD-LL | -25.506 | -0.014 | -23.506 | -0.014 | -2.00 | Linear |
| Eg | ASD-HL | -48.228 | 0.223 | -51.125 | 0.319 | 2.90 | Exponential |
| Eg | TD | -60.210 | 0.308 | -61.912 | 0.357 | 1.70 | ~ |
| Eloc | ASD-LL | -47.965 | 0.092 | -45.965 | 0.092 | -2.00 | Linear |
| Eloc | ASD-HL | -76.978 | 0.323 | -76.662 | 0.337 | -0.32 | ~ |
| Eloc | TD | -77.946 | 0.149 | -77.691 | 0.165 | -0.26 | ~ |
| Cp | ASD-LL | -14.044 | 0.066 | -12.043 | 0.040 | -2.00 | Linear |
| Cp | ASD-HL | -55.575 | 0.244 | -53.934 | 0.224 | -1.64 | ~ |
| Cp | TD | -54.962 | 0.054 | -54.761 | 0.073 | -0.20 | ~ |
| Lp | ASD-LL | 80.841 | -0.014 | 82.841 | -0.014 | -2.00 | Linear |
| Lp | ASD-HL | 28.336 | 0.224 | 25.325 | 0.322 | 3.01 | Exponential |
| Lp | TD | 34.356 | 0.318 | 32.453 | 0.369 | 1.90 | ~ |
| Sigma | ASD-LL | 159.446 | 0.036 | 161.446 | 0.036 | -2.00 | Linear |
| Sigma | ASD-HL | 88.537 | 0.247 | 87.219 | 0.302 | 1.32 | ~ |
| Sigma | TD | 121.054 | 0.286 | 120.979 | 0.305 | 0.08 | ~ |

Note: Eg, global efficiency; Eloc, local efficiency; Cp, clustering coefficient; Lp, characteristic path length; Sigma, small-worldness index. AIC, Akaike Information Criterion; AdjR², adjusted R-squared. ASD-LL, autism spectrum disorder with lower language ability; ASD-HL, autism spectrum disorder with higher language ability; TD, typically developing. Delta AIC = Linear AIC – Exponential AIC. A positive value favors the exponential model, with ΔAIC > 2 indicating a statistically meaningful improvement. “~” denotes that the linear and exponential models provided comparable goodness of fit.

**Table S2.** The shared and distinct nodal hubs across diagnosis groups

| **Hub Category** | **Node Name** | **Full Name** | **MNI Coordinates  (x,y,z)** |
| --- | --- | --- | --- |
|  |  |  |  |
| *Common to ASD-HL & ASD-LL & TD* | | | |
|  | SFGdor.R | Right Superior Frontal Gyrus, dorsolateral | (21.90, 31.12, 43.82) |
|  | PCUN.L | Left Precuneus | (-7.24, -56.07, 48.01) |
|  | PCUN.R | Right Precuneus | (9.98, -56.05, 43.77) |
|  | ITG.L | Left Inferior Temporal Gyrus | (-49.77, -28.05, -23.17) |
| *Common to ASD-HL & TD* | | | |
|  | SPG.R | Right Superior Parietal Gyrus | (26.11, -59.18, 62.06) |
|  | PUT.R | Right Putamen | (27.78, 4.91, 2.46) |
| *Common to ASD-LL & TD* | | | |
|  | FFG.R | Right Fusiform Gyrus | (33.97, -39.10, -20.18) |
| *Unique to ASD-HL* | | | |
|  | SFGdor.L | Left Superior Frontal Gyrus, dorsolateral | (-18.45, 34.81, 42.20) |
| *Unique to TD* | | | |
|  | HIP.R | Right Hippocampus | (29.23, -19.78, -10.33) |
|  | ITG.R | Right Inferior Temporal Gyrus | (53.69, -31.07, -22.32) |

Notes: ASD-HL, autism spectrum disorder with higher language ability; ASD-LL, autism spectrum disorder with lower language ability; TD, typically developing. Regions were identified as hubs if their nodal efficiency exceeded 1 SD above the network mean.

**Table S3.** ANCOVA results based on median splits of five developmental domains in children with ASD

| **Metric** | **Language DQ** | **Personal-social DQ** | **Locomotor DQ** | **Eye-hand coordination DQ** | **Performance DQ** |
| --- | --- | --- | --- | --- | --- |
| Eg | *F=4.06, p=0.02** | *F=2.20, p=0.12* | *F=1.45, p=0.24* | *F=3.94, p=0.02** | *F=2.91, p=0.06* |
| Eloc | *F=0.96, p=0.39* | *F=0.19, p=0.83* | *F=2.50, p=0.09* | *F=0.60, p=0.55* | *F=0.25, p=0.78* |
| Lp | *F=4.35, p=0.02** | *F=2.14, p=0.12* | *F=1.44, p=0.24* | *F=4.24, p=0.02** | *F=3.10, p=0.05* |
| Cp | *F=2.13, p=0.12* | *F=0.46, p=0.63* | *F=2.03, p=0.14* | *F=1.25, p=0.29* | *F=0.74, p=0.48* |
| Lambda | *F=3.63, p=0.03** | *F=1.99, p=0.14* | *F=1.11, p=0.33* | *F=3.19, p=0.05* | *F=2.14, p=0.12* |
| Gamma | *F=2.87, p=0.06* | *F=1.26, p=0.29* | *F=0.63, p=0.54* | *F=2.25, p=0.11* | *F=1.77, p=0.18* |
| Sigma | *F=2.57, p=0.08* | *F=1.10, p=0.34* | *F=0.54, p=0.58* | *F=1.94, p=0.15* | *F=1.62, p=0.20* |
| EdgeNum-Within-L | *F=1.78, p=0.17* | *F=0.88, p=0.42* | *F=0.30, p=0.74* | *F=3.05, p=0.05* | *F=1.67, p=0.19* |
| EdgeNum-Within-R | *F=0.81, p=0.45* | *F=0.48, p=0.62* | *F=0.32, p=0.72* | *F=0.80, p=0.45* | *F=0.63, p=0.54* |
| EdgeNum-Between-L-R | *F=3.48, p=0.03** | *F=3.10, p=0.05* | *F=2.23, p=0.11* | *F=2.77, p=0.07* | *F=2.77, p=0.07* |

Note: Group comparisons were performed using three-group ANCOVA models (Low-DQ ASD, High-DQ ASD, and TD) at a single sparsity threshold (threshold = 3 streamlines), adjusting for age and gender.

**Table S4.** Group ANCOVA comparisons for AUC metrics in FA-weighted network

| **Metric** | **ASD-LL (M±SD)** | **ASD-HL (M±SD)** | **TD (M±SD)** | **F** | **p value** | **Post-hoc p value** | | |
| --- | --- | --- | --- | --- | --- | --- | --- | --- |
|  |  |  |  |  |  | **ASD-LL vs. ASD-HL** | **ASD-LL vs. TD** | **ASD-HL vs. TD** |
| aEg | 0.133 ± 0.005 | 0.136 ± 0.002 | 0.136 ± 0.003 | 6.27 | 0.003 | 0.015 | 0.006 | 0.966 |
| aEloc | 0.177 ± 0.006 | 0.174 ± 0.004 | 0.174 ± 0.004 | 2.95 | 0.057 | 0.097 | 0.11 | 0.995 |
| aLp | 0.472 ± 0.017 | 0.463 ± 0.008 | 0.462 ± 0.008 | 6.49 | 0.002 | 0.013 | 0.005 | 0.97 |
| aCp | 0.123 ± 0.008 | 0.119 ± 0.004 | 0.119 ± 0.004 | 3.66 | 0.029 | 0.059 | 0.062 | 0.997 |
| aGamma | 0.543 ± 0.091 | 0.503 ± 0.049 | 0.500 ± 0.057 | 3.33 | 0.040 | 0.065 | 0.092 | 0.981 |
| aLambda | 0.266 ± 0.005 | 0.263 ± 0.003 | 0.263 ± 0.003 | 5.02 | 0.008 | 0.022 | 0.023 | 0.998 |
| aSigma | 0.509 ± 0.075 | 0.478 ± 0.042 | 0.475 ± 0.048 | 2.96 | 0.056 | 0.086 | 0.122 | 0.979 |

**Figure S1. Validation analysis of global network topology using fractional anisotropy (FA)-weighted structural networks.** Left panels show sparsity-dependent profiles of global network metrics, including global efficiency (Eg), local efficiency (Eloc), characteristic path length (Lp), clustering coefficient (Cp), and small-worldness (Sigma), across a sparsity range of 10% to 35% in 1% increments. Asterisks indicate sparsity thresholds showing significant group effects among the three subgroups (ASD-LL, ASD-HL, and TD; *p* < 0.05). Right panels show violin plots of the integrated area under the curve (AUC) for each corresponding network metric across the 10%–35% sparsity range.

**Figure S2. Correlation analyses between FA-weighted network metric and clinical behavior scores in children with ASD.** Scatter plots illustrating the partial correlations (controlling for age and gender) between global efficiency AUC (aEg) and autism symptom severity (ABC scores; top row) and Language developmental quotients (Language DQ; bottom row) across the ASD cohorts: (left) the low-language autism subgroup (ASD-LL), (middle) the high-language autism subgroup (ASD-HL), and (right) the combined ASD group (ASD combined). Significant correlation coefficients (*p* < 0.05) are presented in bold red text.
